# Comparative Transcriptome and Volatile Metabolome Analysis of *Gossypium hirsutum* Resistance to Verticillium Wilt

**DOI:** 10.3390/genes16080877

**Published:** 2025-07-25

**Authors:** Ni Yang, Chaoli Xu, Yajun Liang, Juyun Zheng, Shiwei Geng, Fenglei Sun, Shengmei Li, Chengxia Lai, Mayila Yusuyin, Zhaolong Gong, Junduo Wang

**Affiliations:** 1 National Cotton Engineering Technology Research Center, Cotton Research Institute of Xinjiang Uygur Autonomous Region Academy of Agricultural Sciences, Urumqi 830091, China13999966149@163.com (Y.L.);; 2 Xinjiang Agricultural Vocational and Technical University, Changji 831100, China

**Keywords:** RNA-seq, GC–MS, amino acids, anthocyanins, WGCNA

## Abstract

Background: In recent years, changes in climate conditions and long-term continuous cropping have led to the increased occurrence of Verticillium wilt in various cotton-growing regions, causing significant economic losses in cotton production. Research has shown that volatile substances are closely linked to plant disease resistance; however, studies on their roles in the response of cotton to Verticillium wilt, including their relationship with gene regulation, are limited. Methods: In this study, the transcriptomes and metabolomes of Xinluzao 57 (a highly susceptible Verticillium wilt variety) and 192,868 (a highly resistant Verticillium wilt variety) were sequenced at different time points after inoculation with Verticillium wilt. Results: A total of 21,911 commonly differentially expressed genes (DEGs) were identified within and between the materials, and they were clustered into eight groups. Significant annotations were made in pathways related to amino acids and anthocyanins. Metabolomics identified and annotated 26,200 volatile metabolites across nine categories. A total of 158 differentially accumulated metabolites (DAMs) were found within and between the materials; three clusters were identified, and the 10 metabolites with the most significant fold changes were highlighted. Weighted gene coexpression network analysis (WGCNA) revealed that 13 genes were significantly correlated with guanosine, 6 genes were correlated with 2-deoxyerythritol, and 32 genes were correlated with raffinose. Conclusions: Our results provide a foundation for understanding the role of volatile substances in the response of cotton to Verticillium wilt and offer new gene resources for future research on Verticillium wilt resistance.

## 1. Introduction

Cotton has become one of the most important fiber crops worldwide. It is one of the most important raw materials in the textile industry, defense industry, and pharmaceutical industry, accounting for approximately 25% of the world’s total fiber use [1]. *Gossypium hirsutum* is the cotton variety with the largest planting area worldwide, and its output accounts for approximately 97% of the world’s cotton output [2]. Owing to the lack of Verticillium wilt-resistant varieties in production, Verticillium wilt has become one of the important factors restricting the production of *G. hirsutum*, thereby severely hindering improvements in cotton yield and fiber quality [3]. Verticillium wilt fungi infect plants from the roots; after colonization in the roots, they spread to the entire plant via the vascular tissues, thus causing the cotton leaves to dehydrate, lose greenness, and wilt, which can cause plant death in severe cases [4]. Verticillium wilt can affect more than 200 dicotyledonous plants, including annual herbaceous, perennial, and woody plants. In leading cotton-producing nations such as China, India, the United States, and Pakistan, the typical reduction in cotton crop yields due to Verticillium wilt ranges from 10 to 35% [5].

On the basis of systems biology, multiomics joint analysis emphasizes the interrelationships and integrity between different levels in organisms, which aids in revealing the complexity and interactive network of biological systems [6]. The integration of transcriptomics and metabolomics has been instrumental in studying plant growth, development, and response to stress. This approach is commonly employed to elucidate the genetic and regulatory processes underlying plant metabolites [7,8]. Currently, the analysis of volatile substances is mainly determined via gas chromatography–mass spectrometry (GC–MS) [9]. GC–MS technology integrates the precise separation abilities of gas chromatography with the accurate identification capabilities of mass spectrometry, allowing for the quantitative analysis of volatile compounds [10]. Moreover, this method is characterized by high sensitivity, simple operation, and authenticity. Transcriptomics is used to investigate gene transcription and regulation as a whole and is often used to analyze key regulatory genes involved in plant growth, resistance, and metabolism [11,12]. For example, transcriptomic analysis of TM-1 and Hai7124 at various time points after inoculation with Verticillium wilt revealed *MYB14* as a key player in conferring resistance to the disease in cotton [13]. Moreover, transcriptome and metabolome analyses of the three periods of inoculation with TM-1 and Hai7124 revealed that the glutathione metabolic pathway plays an important role in resisting Verticillium wilt; additionally, *glutathione S-transferase (GSTF8)* was identified as an important candidate gene involved in this pathway [14]. The joint analysis of transcriptomics and metabolomics identified five key metabolites in the phenylpropanoid biosynthesis pathway, such as caffeic acid, coniferyl alcohol, coniferin, and scopolamine, that exhibit potent inhibitory effects on the growth of Verticillium wilt. These findings suggest a potential relationship between these metabolites and cotton resistance to disease [15]. However, research on the regulation of volatile substances and genes in cotton during pathogen infection is still limited.

Currently there is a lack of effective prevention and control measures for Verticillium wilt during cotton production. The development of resistant cotton varieties through breeding is a practical, cost-effective, and sustainable approach to combatting Verticillium wilt. This strategy is crucial for promoting the continued growth and health of the cotton industry [3]. However, owing to the lack of excellent resistance sources in *G. hirsutum*, it has become very difficult to breed disease-resistant varieties [16]. Therefore, the exploration of new disease resistance mechanisms and genes is very important for breeding *G. hirsutum* resistant to Verticillium wilt. Recent studies have increasingly demonstrated the considerable involvement of volatile substances in plant disease resistance [17,18]. Currently, many reports on cotton omics research exist; however, most of these studies have focused on fiber quality and coping mechanisms for abiotic stress [19,20]. Moreover, relatively few studies have investigated the response mechanisms to Verticillium wilt (mainly the composition, content, and regulatory network of volatile substances), and the role of volatile substances in the response to Verticillium wilt has not been analyzed. Therefore, this study conducted GC–MS and RNA-seq sequencing on *G. hirsutum* strains resistant to Verticillium wilt using materials inoculated with the fungus over four time periods. Using bioinformatics analysis, we investigated the response of *G. hirsutum* to Verticillium wilt via transcriptomics and metabolomics, shedding light on the gene-metabolite regulatory network involved in volatile substance production. Understanding the role of volatile substances in the defense of *G. hirsutum* against Verticillium wilt offers molecular insights and enhances our understanding of their functions, potentially providing guidance for processing and utilization.

## 2. Materials and Methods

### 2.1. Plant Material

In this study, we used the highly susceptible variety Xinlu Zao 57 (Xinlu Zao 57 is an early maturing *G. hirsutum* variety bred by the Economic Crops Research Institute of Xinjiang Academy of Agricultural Sciences; it passed Xinjiang Approval in 2013). The highly resistant line 192868 (192868 is a new line of *G. hirsutum* with high resistance to Verticillium wilt bred by the Economic Crops Research Institute of Xinjiang Academy of Agricultural Sciences, unapproved), which was provided by the Institute of Economic Crops of Xinjiang Academy of Agricultural Sciences, was also selected for use. In February 2024, indoor experiments were conducted at the Xinjiang Academy of Agricultural Sciences. The seedling medium was prepared by mixing the soil and vermiculite at a 2:1 ratio, after which the mixture was sterilized at 121 °C for 20 min. High-quality seeds were carefully selected, disinfected with 5% sodium hypochlorite for 5 min and 75% alcohol for 30 s, rinsed thoroughly with sterile water, and placed on filter paper in a glass culture dish. The dish was then incubated at a temperature of 24–28 °C for germination. The germinated seeds were subsequently transplanted into a 32-well plug tray filled with seedling medium and transferred to a greenhouse for further cultivation. The seedlings were grown under controlled conditions of 25–30 °C, 4000 lx light intensity, and a photoperiod of 16 h of light, followed by 8 h of darkness. Cotton seedlings with 3 true leaves were selected and uniformly inoculated with the highly pathogenic strain Verticillium dahliae Vd-3 via the root tearing method. Samples were taken at 0, 24, 48, and 72 h after inoculation. Each sample had 10 biological replicates, with 3 replicates for RNA-seq, 6 replicates for metabolome sequencing, and 3 replicates for qRT–PCR analyses.

### 2.2. RNA-Seq and Analysis

RNA extraction, library construction, and sequencing were completed by Xinjiang Aidesen Biotechnology Co., Ltd. (Urumqi, China). RNA extraction was carried out using a polysaccharide and polyphenol plant total RNA extraction kit, ensuring that each sample consisted of approximately 100 mg. The extraction protocol followed the manufacturer’s guidelines. The purity (OD260/280) and concentration of the RNA samples were subsequently assessed using a Nanodrop spectrophotometer to verify the normal nucleic acid absorption peaks. RNA integrity was assessed using an Agilent 2100 instrument by analyzing the RIN value, the 28S/18S ratio, baseline changes in the spectrum, and the presence of the 5S peak. Once the RNA samples passed the quality assessment, they were sequenced via Illumina HiSeq 2500 technology. Fastp software (V0.23.4) was subsequently used to eliminate adapter sequences and filter out low-quality reads and sequences with N ratios exceeding 5%, generating clean reads suitable for downstream analysis [21]. HISAT2 was used to align the clean reads to the reference genome of TM-1 (https://www.CottonGen.org/species/Gossypium_hirsutum/ZJU-AD1_v2.1, accessed on 28 February 2025) [22]. The results were statistically analyzed and quantified via featureCounts [23]. Unnormalized read count data were used as input data to calculate *p* values and fold changes using DESeq2 software (version 1.48.1), and FDR < 0.01 and |log2fold change| > 1 were used as screening criteria to obtain the DEGs [24]. Enrichment analyses of the DEGs via Gene Ontology (GO) and Kyoto Encyclopedia of Genes and Genomes (KEGG) analyses were conducted using the clusterProfiler software (version 4.16.0) package in R [25].

### 2.3. Gas Chromatography–Mass Spectrometry Sequencing and Analysis

A CTC trinity autosampler was used with the following conditions: extraction head at 50/30 μmDVB/CARonPDMS, temperature at 50 °C, oscillation time of 15 min, extraction time of 30 min, oscillation speed of 250 rpm, analysis time of 5 min, and GC cycle time of 50 min. The analysis was conducted using an Agilent 7890 gas chromatograph coupled with a LECO PEGASUS HT high-resolution mass spectrometer. The samples were analyzed under the following conditions. The derivatized compounds were separated via a constant flow of helium at 1 mL/min. The sample was introduced into a 20 mL headspace bottle, which was sealed and capped. The injection port temperature was set to 260 °C, and the heating program was initiated at 40 °C for 5 min, followed by an increase to 220 °C at a rate of 5 °C/min, a further increase to 250 °C at a rate of 20 °C/min, and maintenance for 2.5 min. Mass spectrometry data were processed using ChromaTOF software (version 4.30), which utilizes the LECO-Fiehn Rtx5 database for quantitative metabolite analysis, including the matching of mass spectra and retention time indices. Correlation analysis and principal component analysis (PCA) were conducted on each sample via the R programming language on the basis of the metabolite content data matrix [26]. Differentially abundant metabolites were identified via criteria such as a fold change ≥ 2, a fold change ≤ 0.5, and a *p* value < 0.05.

### 2.4. Weighted Gene Coexpression Network Analysis

The R language WGCNA package was used to conduct coexpression analysis of the gene expression profiles of the differentially expressed genes (DEGs) using the dynamic branch cutting method [27]. A weighting coefficient of β = 12 was chosen for the analysis. The network construction was carried out using the blockwiseModules function, resulting in the identification of several distinct modules with varying numbers of genes within each module. Modules with a similarity of 0.75 were merged using minModuleSize = 30 and Merge Cut Height = 0.25 as the criteria. The correlation coefficients between the module eigengene (ME) and hormone levels at different treatment time points were computed. The coexpression network was visualized using Cytoscape software (version 3.10.1) [28].

### 2.5. Quantitative Real-Time Polymerase Chain Reaction

Gene-specific primers were designed using Primer 5.0 software on the basis of cDNA sequences. The total RNA was extracted in February 2024 using the RNAprep Pure Polysaccharide and Polyphenol Plant Total RNA Extraction Kit (Tiangen, China), with each sample subjected to triplicate analyses. The RNA concentration was determined using a NanoDrop 2000 spectrophotometer (Thermo Fisher Scientific, Waltham, MA, USA). Reverse transcription was carried out using the M-MLV RTase cDNA Synthesis Kit (TaKaRa, Japan) to generate cDNA. Real-time PCR amplification was conducted on a Bio-Rad CFX96 Real-time System. The iTaq Universal SYBR Green Supermix Kit from Bio-Rad (USA) was utilized following the manufacturer’s instructions, with a total reaction volume of 20 μL. The amplification protocol consisted of an initial denaturation step at 95 °C for 30 s, followed by denaturation at 95 °C for 5 s, annealing at 57 °C for 5 s, and extension at 72 °C for 34 s, which was repeated for 40 cycles. The results were analyzed through relative quantitative analysis using the 2^−ΔΔCt^ method, with the internal reference gene GhUBQ7 [29].

## 3. Results

### 3.1. RNA-Seq Global Analysis of the Response of G. hirsutum to Verticillium Wilt

**Figure 1:**
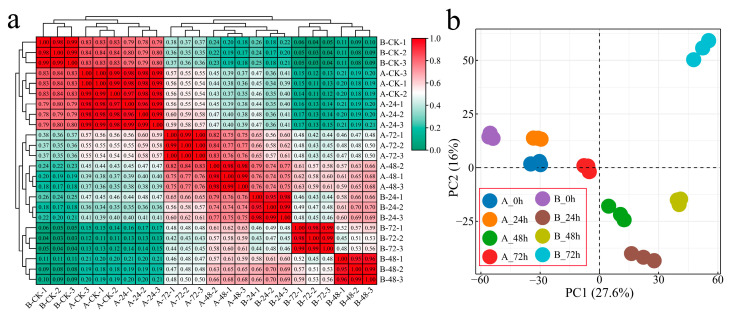
Clustering and PCA of the RNA-seq data. (**a**) Heatmap and cluster analysis of the RNA-seq data from Verticillium wilt inoculated with *G. hirsutum* (A: Xinluzao 57, B: 192868) and (**b**) PCA of the RNA-seq data from *G. hirsutum* inoculated with Verticillium wilt (A: Xinluzao 57, B: 192868).

After sequencing for quality control, 24 samples of two *G. hirsutum* plants inoculated with Verticillium wilt at four stages were sequenced, and 166.84 Gb of clean data was obtained. Each sample achieved a Q30 score of over 94.32%, and the alignment efficiency of the reads to the reference genome ranged from 82.93 to 89.79% (Table S2). Correlation analysis was conducted on the RNA-seq data from the 24 samples, with correlation coefficients exceeding 0.95 among biological replicates of the same sample, indicating reliable and reproducible transcriptome data (Figure 1a). To study the transcriptome changes in *G. hirsutum* in response to Verticillium wilt, hierarchical clustering and principal component analysis (PCA) were performed on samples collected at four different stages from the two plant materials (Figure 1a,b). At 0 h after inoculation, the distance between the two materials was the shortest, and the greatest correlation was observed. The distance between the samples of the susceptible variety Xinluzao 57 after inoculation with Verticillium wilt was relatively close, and the closest correlation of the clusters of the samples at 0 and 24 h after inoculation was greater than 0.97. However, the distance between the samples of the disease-resistant strain 192868 after inoculation with Verticillium wilt was large, and the correlation coefficient was small. The difference between the materials was greater than the difference within the materials, indicating that the material selected was reasonable and that samples subjected to the same treatment were clustered together, confirming the reliability and repeatability of the RNA-seq data.

**Figure 2:**
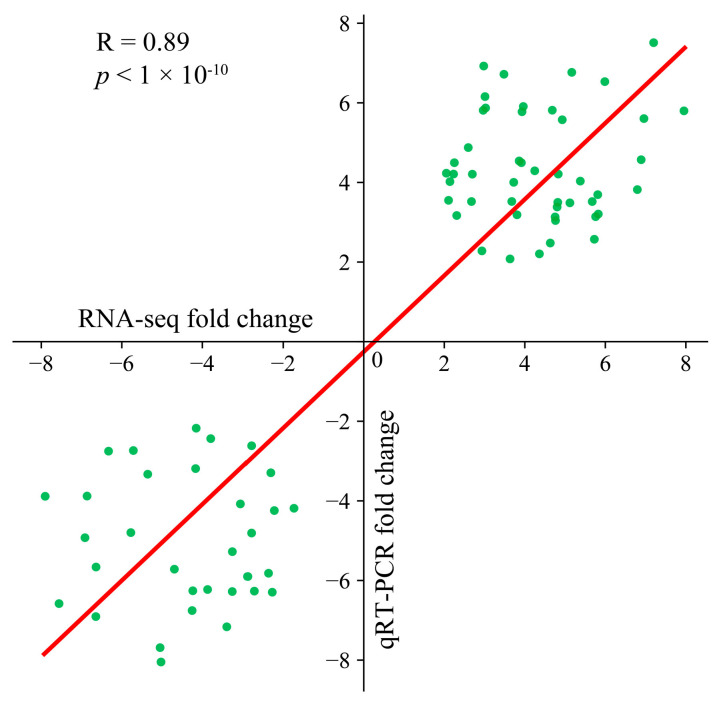
Scatter plot of the correlation and significance of the RNA-seq and qRT–PCR results.

Ten genes were randomly chosen for three separate qRT–PCR analyses to confirm the consistency of the transcriptome data. The transcriptome data of these selected genes were strongly correlated with the fold change observed in the qRT–PCR results (R = 0.88, *p* < 0.01), confirming the reliability of the transcriptome sequencing data for subsequent analyses (Figure 2).

### 3.2. Differential Expression Analysis

**Figure 3:**
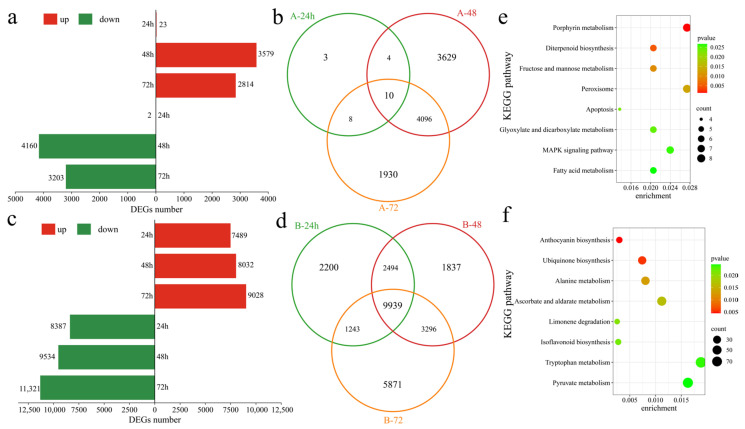
Differential expression analysis. (**a**) Numbers of upregulated and downregulated DEGs at different time points after Xinluzao 57 inoculation, (**b**) Venn diagram of DEGs at different time points after Xinluzao 57 inoculation (A: Xinluzao 57), (**c**) numbers of upregulated and downregulated DEGs at different time points after 192868 inoculation, (**d**) Venn diagram of DEGs at different time points after 192868 inoculation (B: 192868), (**e**) KEGG enrichment analysis of the DEGs of only Xinluzao 57, and (**f**) KEGG enrichment analysis of the DEGs of only 192868.

To assess the transcriptional differences between the two varieties in response to Verticillium wilt, differentially expressed genes (DEGs) in the susceptible variety Xinluzao 57 were identified at various time points after inoculation. At 24 h post inoculation, compared with those at the pre-inoculation stage, 25 DEGs were recognized, with 23 upregulated and 2 downregulated (Figure 3a). At 48 h post inoculation, 7739 DEGs were detected, with 3579 upregulated and 4160 downregulated. Similarly, at 72 h post inoculation, 6044 DEGs were identified, including 2841 upregulated and 3203 downregulated genes. In the susceptible variety Xinluzao 57, 9680 DEGs were found after inoculation, with 3 unique DEGs at 24 h, 3629 unique DEGs at 48 h, and 1930 unique DEGs at 72 h, along with 10 common DEGs. Conversely, in the resistant strain 192868, 26,880 DEGs were identified at different time points after inoculation. Specifically, at 24 h post inoculation, 15,876 DEGs were observed, with 7489 upregulated and 8387 downregulated genes (Figure 3c). Moreover, 17,566 DEGs were identified at 48 h post inoculation, with 8032 upregulated and 9534 downregulated genes. Furthermore, 72 h after inoculation, 20,349 DEGs were detected, including 9028 upregulated and 11,321 downregulated genes. Among these, there were 2200 unique DEGs at 24 h, 1837 unique DEGs at 48 h, and 5873 unique DEGs at 72 h. Additionally, 9939 DEGs were common across all time points (Figure 3d). Further analysis revealed 1200 unique DEGs in Xinluzao 57 and 18,400 unique DEGs in 192868 (Figure S1). To further identify the functions and regulatory mechanisms of these specific DEGs, KEGG enrichment analysis was performed. Xinluzao 57 contained 1200 unique DEGs that were significantly annotated in the porphyrin metabolism, diterpenoid biosynthesis, fructose and mannose metabolism, peroxisome, apoptosis, glyoxylate and dicarboxylate metabolism, and MAPK signaling and fatty acid metabolism pathways (Figure 3e). Furthermore, 18,400 unique DEGs in 192868 were significantly annotated in anthocyanin biosynthesis, ubiquinone biosynthesis, alanine metabolism, ascorbate and aldarate metabolism, limonene degradation, isoflavonoid biosynthesis, and the tryptophan metabolism and pyruvate metabolism pathways (Figure 3f).

**Figure 4:**
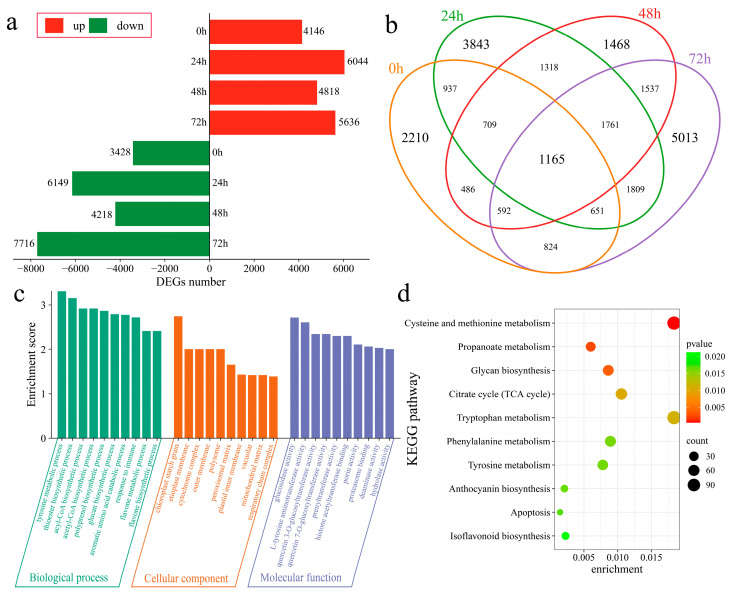
Differential expression analysis among plant materials. (**a**) Numbers of upregulated and downregulated DEGs at the same time points after inoculation between Xinluzao 57 and 192868, (**b**) Venn diagram of DEGs at the same time points after inoculation between Xinluzao 57 and 192868, (**c**) GO enrichment analysis of DEGs at the same time points after inoculation between Xinluzao 57 and 192868, and (**d**) KEGG enrichment analysis of DEGs at the same time points after inoculation between Xinluzao 57 and 192868.

The DEGs in Xinluzao 57 and 192868 at corresponding time points after inoculation were further analyzed. At 0 h after inoculation, 7574 DEGs were identified, with 4146 upregulated and 3428 downregulated genes (Figure 4a). At 24 h after inoculation, 13,193 DEGs were subsequently detected, including 6044 upregulated and 6149 downregulated genes. Additionally, 9036 DEGs were found at 48 h post inoculation, with 4818 upregulated and 4218 downregulated genes. Furthermore, at 72 h after inoculation, 13,352 DEGs were identified, including 5636 upregulated and 7716 downregulated genes. A total of 24,323 DEGs were identified in the plant material at the corresponding time points after inoculation, including 2210 unique DEGs at 0 h, 3843 unique DEGs at 24 h, 1468 unique DEGs at 48 h, and 5013 unique DEGs at 72 h. Additionally, 1165 common DEGs were detected across all time points (Figure 4b). To further identify the functions and regulatory mechanisms of these DEGs, GO and KEGG enrichment analyses were performed. GO enrichment analysis significantly annotated the biological processes of the tyrosine metabolic process, thioester biosynthetic process, acyl-CoA biosynthetic process, acetyl-CoA biosynthetic process, polyprenol biosynthetic process, glucan biosynthetic process, aromatic amino acid catabolic process, immune response process, flavone metabolic process, and flavone biosynthetic process (Figure 4c). KEGG analysis revealed significant enrichment in cysteine and methionine metabolism, propanoate metabolism, glycan biosynthesis, the citrate cycle (TCA cycle), tryptophan metabolism, phenylalanine metabolism, tyrosine metabolism, anthocyanin biosynthesis, apoptosis, and isoflavonoid biosynthesis pathways (Figure 4d).

**Figure 5:**
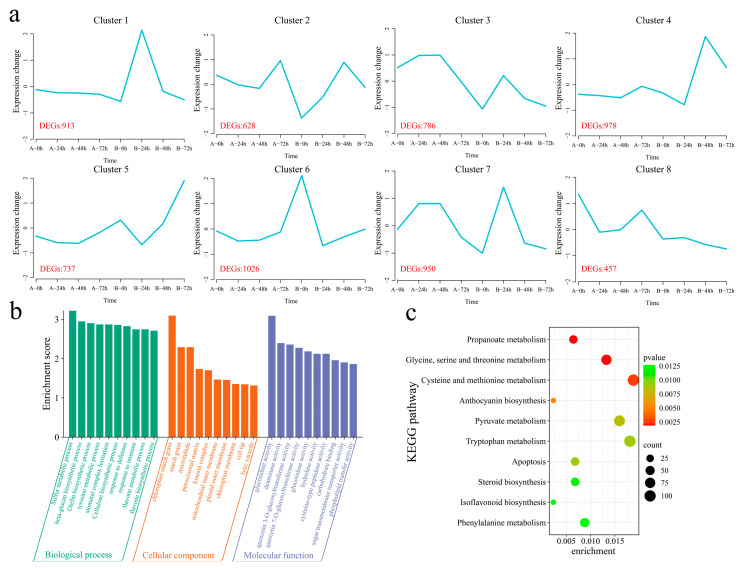
K-means clustering and enrichment analysis of common DEGs within and between the plant materials. (**a**) K-means clustering line graph of the DEGs (A: Xinluzao 57, B: 192868), (**b**) GO annotation of DEGs, and (**c**) KEGG pathway annotation of DEGs.

A total of 21,911 common DEGs were identified within and between the materials (Figure S2). The k-means clustering algorithm was used to cluster the 21,911 DEGs into eight clusters (Figure 5a). Cluster 1 (which included 913 DEGs) presented the highest expression level in the resistant strain 192868 24 h after inoculation, and the expression level remained essentially unchanged in the susceptible variety Xinluzao 57. Cluster 2 (including 628 DEGs) presented the maximum expression level 48 h after inoculation in the resistant strain 192868, with the expression level decreasing in the susceptible variety Xinluzao 57 after inoculation and increasing at 72 h. Cluster 3 (including 786 DEGs) showed the same expression trend in the two plant materials, with the expression levels of both plants tending to increase and subsequently decrease after inoculation; however, the expression level in the resistant strain 192868 was lower than that in the susceptible variety Xinluzao 57. Cluster 4 (which included 978 DEGs) reached its maximum expression level 48 h after inoculation of the resistant strain 192868, and this level remained essentially unchanged in the susceptible variety Xinluzao 57. The expression trends of Cluster 5 (including 737 DEGs) were consistent in both materials, with the levels in both plants increasing after inoculation; however, the expression level in the resistant strain 192868 was greater than that in the susceptible variety Xinluzao 57. The expression levels of Cluster 6 genes (including 1026 DEGs) decreased after inoculation with the resistant strain 192868, but remained essentially unchanged in the susceptible variety Xinluzao 57. Cluster 7 (which included 950 DEGs) reached its maximum expression level 48 h after inoculation of the resistant strain 192868, with this level initially increasing but then decreasing in the susceptible variety Xinluzao 57, whereas it reached its maximum value at 24 h. Cluster 8 (which included 457 DEGs) reached its minimum expression level 72 h after inoculation with the resistant strain 192868, with the expression level initially decreasing but subsequently increasing in the susceptible variety Xinluzao 57. To further reveal the functions of the 21,911 common DEGs that were identified within and between the plant materials, we performed GO and KEGG enrichment analyses. GO enrichment analysis revealed significant annotations related to various biological processes, including the sulfur metabolic process, beta-glucan biosynthetic process, olefin biosynthetic process, tyrosine metabolic process, stomatal complex formation process, cellulose biosynthetic process, defense response process, immune response process, flavone metabolic process, and flavone biosynthetic process (Figure 5b). KEGG analysis revealed significant enrichment in propanoate metabolism; glycine, serine, and threonine metabolism; cysteine and methionine metabolism; anthocyanin biosynthesis; pyruvate metabolism; tryptophan metabolism; apoptosis; steroid biosynthesis; isoflavonoid biosynthesis; and phenylalanine pathways (Figure 5c).

### 3.3. Metabolome Analysis

**Figure 6:**
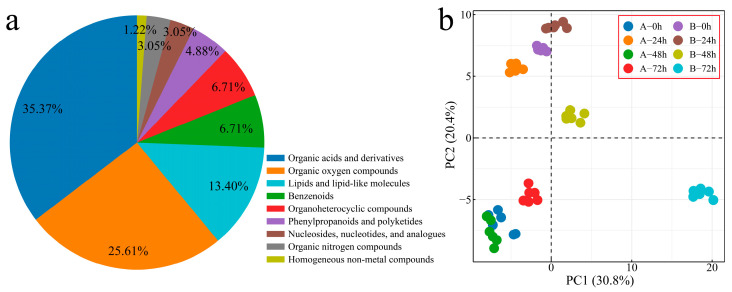
PCA of overall metabolite classification. (**a**) Pie chart of overall metabolite classification proportions and (**b**) PCA of overall metabolites (A: Xinluzao 57, B: 192868).

To explore the changing characteristics of volatile substances during the process of Verticillium wilt inoculation of *G. hirsutum*, we performed metabolome sequencing on 48 samples from two plant materials at four inoculation periods (with six biological replicates included for each sample), and 200 volatile metabolites were detected. The 200 identified metabolites were classified and annotated, and the annotated metabolites were mainly divided into 9 categories (Figure 6a). Organic acids and their derivatives accounted for 35.37% of the total number of identified metabolites, with organic oxygen compounds accounting for 25.61% and lipids and lipid-like molecules accounting for 13.41%. The correlation analysis and PCA of the samples revealed that the correlation between the replicates was good (r > 0.90), and that the different biological replicates of the same sample were clustered together, indicating that the error between the replicates was small and that the sample quality was good. Thus, the next step of analysis could be performed (Figure 6b and Figure S3).

### 3.4. Differentially Accumulated Metabolite (DAM) Analysis

**Figure 7:**
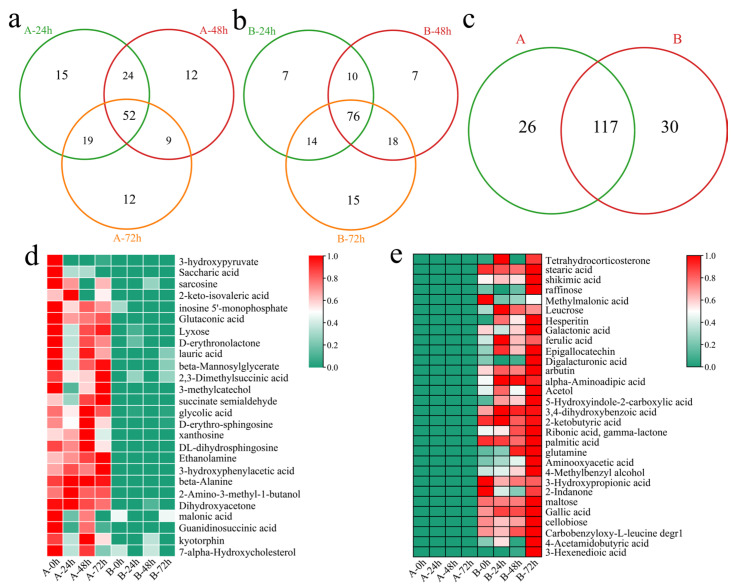
Identification of DAMs. (**a**) Venn diagram of DAMs at different time points after inoculation of Xinluzao 57 (A: Xinluzao 57), (**b**) Venn diagram of DAMs at different time points after inoculation of 192868 (B: 192868), (**c**) Venn diagram of specific and common DAMs at different time points after inoculation of Xinluzao 57 and 192868 (A: Xinluzao 57, B: 192868), (**d**) heatmap of the contents of specific DAMs in Xinluzao 57, and (**e**) heatmap of the contents of specific DAMs in 192868.

To investigate the transcriptional differences between the two varieties in response to Verticillium wilt, differentially accumulated metabolites (DAMs) were initially identified in the susceptible variety Xinluzao 57 at different time points after inoculation. Compared with those before inoculation, 110 DEGs were identified 24 h after inoculation, including 15 unique DAMs (Figure 7a). A total of 97 DAMs (including 12 unique DAMs) were identified 48 h after inoculation. Moreover, 92 DAMs (including 12 unique DAMs) were identified 72 h after inoculation. In total, 143 DAMs (including 52 common DEGs) were identified at different time points after inoculation in Xinluzao 57. In the resistant line 192868, 107 DAMs were identified at 24 h after inoculation, including 7 unique DAMs (Figure 7b). Additionally, 111 DAMs were identified 48 h after inoculation, including 7 unique DAMs. A total of 123 DAMs (including 15 unique DAMs) were identified 72 h after inoculation. Furthermore, 147 DAMs were identified, including 76 common DAMs. Further analysis revealed that 26 unique DAMs were detected in Xinlu Zao 57 and that 30 unique DAMs were detected in 192868 (Figure 7c). The specific DAMs detected in Xinlu Zao 57 were present at the highest levels at 0, 48, and 72 h, among which the 3-hydroxypyrene and saccharic acid contents decreased after inoculation (Figure 7d). The specific DAMs identified in 192868 were present at the highest levels at 72 h, among which the contents of methylmalonic acid, 3-hydroxypropionic acid, and 2-indanone decreased after inoculation (Figure 7e).

**Figure 8:**
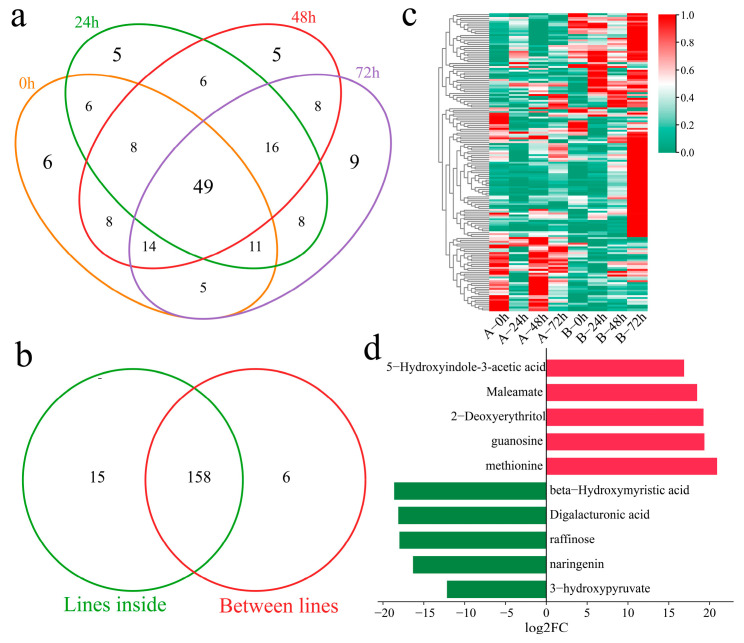
Analysis of DAMs between the plant materials. (**a**) Venn diagram of DAMs of Xinluzao 57 and 192868 inoculated at the same time points, (**b**) Venn diagram of specific and shared DAMs within and between the materials, (**c**) cluster heatmap of shared DAMs within and between the materials, and (**d**) log2-fold changes in the top 10 metabolites.

The DAMs of Xinluzao 57 and 192868 at the same time points of inoculation were further identified. A total of 107 DAMs were identified at 0 h, including six unique DEGs (Figure 8a). Additionally, 109 DAMs were identified at 24 h after inoculation, including five unique DEGs. A total of 114 DAMs were identified at 48 h after inoculation, including five unique DEGs. Moreover, 120 DAMs were identified at 72 h after inoculation, including nine unique DEGs. A total of 165 DAMs were identified at the same time points between the plant materials, including 49 common DAMs. In addition, 158 common DAMs were identified within and between the materials (Figure 8b). A total of three clusters were identified for the 158 DAMs within and between the materials via k-means clustering (Figure 8c). Cluster 1 presented the highest content 24, 48, and 72 h after inoculation in the resistant strain 192868, and the content was essentially unchanged in the susceptible variety Xinluzao 57. Cluster 2 presented the highest content 72 h after inoculation in the resistant strain 192868, and the content was essentially unchanged in the susceptible variety Xinluzao 57. The content of Cluster 3 was essentially unchanged in the resistant strain 192868, and the highest content was detected 0 and 48 h after inoculation in the susceptible variety Xinluzao 57. The ten metabolites exhibiting the greatest fold changes were further identified, among which the expression levels of 5-hydroxyindole-3-acetic acid, maleamate, 2-deoxyerythritol, guanosine, and methionine were mainly upregulated, with log2-fold changes in absolute values greater than 18. Moreover, the expression levels of beta-hydroxymyristic acid, digalacturonic acid, raffinose, naringenin, and 3-hydroxypyruvate were downregulated, and their log2-fold changes in absolute values were above 12.

### 3.5. WGCNA

**Figure 9:**
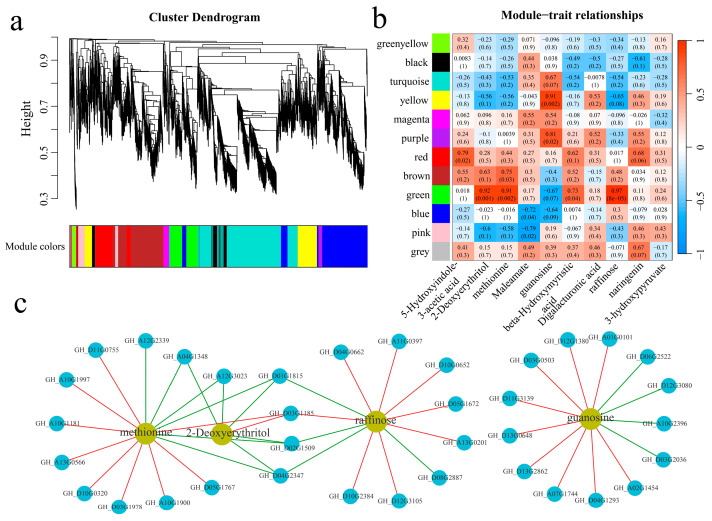
WGCNA. (**a**) WGCNA clustering dendrogram; different colors represent different modules, (**b**) module and key metabolite correlation and significance analysis heatmap, and (**c**) metabolite and gene correlation network diagram; the red line represents a positive correlation, and the green line represents a negative correlation.

A total of 21,911 common DEGs were identified within and between the plant materials. Twelve coexpression modules were obtained via WGCNA (Figure 9a). The correlation and significance between the modules and the 10 metabolites exhibiting the largest fold changes were calculated (Figure 9b). The yellow and purple modules were significantly highly correlated with guanosine, and the green module was significantly highly correlated with 2-deoxyerythritol, methionine, and raffinose. To further explore the relationships between the module genes and metabolites, the correlations between the genes and metabolites were calculated, and the absolute values of the correlation coefficients were above 0.90, with *p* values of less than 0.05 for visualization (Figure 9c). Thirteen genes were significantly correlated with guanosine; specifically, four genes were significantly negatively correlated with guanosine, and nine genes were significantly positively correlated with guanosine. Moreover, six genes were significantly correlated with 2-deoxyerythritol; specifically, five genes were significantly negatively correlated with 2-deoxyerythritol, and one gene was significantly positively correlated with 2-deoxyerythritol. Fifteen genes were significantly correlated with methionine; specifically, six genes were significantly negatively correlated with methionine, and nine genes were significantly positively correlated with methionine. Moreover, twelve genes were significantly correlated with raffinose; specifically, four genes were significantly negatively correlated with raffinose, and eight genes were significantly positively correlated with raffinose. GH_A04G1348 and GH_A12G3023 were significantly negatively correlated with 2-deoxyerythritol and methionine. Additionally, GH_D01G1815, GH_D02G1509, and GH_D04G2347 were significantly negatively correlated with 2-deoxyerythritol, methionine, and raffinose. Furthermore, GH_D03G1185 was significantly positively correlated with 2-deoxyerythritol, methionine, and raffinose.

## 4. Discussion

As a raw material for the textile industry, cotton is affected by various biological and abiotic stresses during its growth, including various pests, pathogens, high temperatures, low temperatures, and drought, which can lead to severe losses in cotton yield and quality [30]. In recent years, with changes in climatic conditions and long-term continuous cropping activities, Verticillium wilt has frequently occurred in various cotton-growing areas, causing severe global economic losses in cotton production [31]. Plant volatiles have received extensive attention in the study of ecology and herbivorous insects, and an increasing number of studies have demonstrated that such substances are related to plant disease resistance [32,33]. However, there is currently a lack of research on changes in volatile substances in cotton in response to Verticillium wilt, especially regarding the relationship between these substances and gene regulation. Therefore, investigating the regulatory mechanism between volatile substances and genes in response to Verticillium wilt in cotton is highly important. Therefore, we selected the highly resistant Verticillium wilt line 192868 and the highly susceptible Verticillium wilt variety Xinlu Zao 57 for analysis of the gas-phase metabolome combined with transcriptome sequencing at different stages of inoculation with Verticillium wilt. PCA, clustering analysis, and differential expression analysis of the RNA-seq data revealed that the disease-resistant line 192868 presented more DEGs and smaller correlation coefficients between the samples after inoculation with Verticillium wilt. In the metabolomics dataset, the number of DAMs slightly differed between the Verticillium wilt-resistant line 192868 and the highly susceptible variety Xinluzao 57. These findings indicate that some metabolites are ineffective in the defense responses of cotton in response to Verticillium wilt.

In plants, flavonoid metabolites are crucial in the plant response to pathogen infection [34]. Flavonoid metabolites can participate in plant defense against pathogens by removing reactive oxygen species (ROS) and activating defense pathways, thus improving plant resistance to pathogens [35,36]. Flavonoid compounds can inhibit spore germination and the mycelial growth of plant pathogens. Studies have shown that apigenin, meditartin, 7,4′-dihydroxyflavone, naringenin, and daidzein in alfalfa exert significant inhibitory effects on the response to FW [37]. Phlorizin can increase the activity of defense-related enzymes in apples and promote the accumulation of resistant substances (such as total phenols, flavonoids, and lignin) while reducing the content of H_2_O_2_ [38]. Additionally, the overexpression of flavonoid biosynthesis genes in sorghum can reduce the oxidative damage caused by anthracnose. In soybeans, *GmF3H1*, *GmF3H2*, and *GmFNSII-1* can improve resistance to mosaic virus by regulating the increase in the content of flavonoid pathway metabolites [39]. The enrichment analysis of the DEGs revealed that the biosynthesis pathways of flavonoids and phenylpropanoids were significantly enriched in the disease-resistant line 192868. These findings indicate that the flavonoid pathway plays an important role in the resistance of *G. hirsutum* to Verticillium wilt and that several genes in the flavonoid pathway were upregulated. The fold change in naringenin in the flavonoid pathway exceeded an absolute value of 15 after cotton was invaded by Verticillium wilt, and naringenin exhibited strong antioxidant activity in plants. These findings indicate that naringenin in the flavonoid pathway may play an important role in the response of cotton to Verticillium wilt.

Plant volatile substances usually have certain functions in regulating the interactions among plants, microorganisms, and insects; moreover, pathogen infection can cause the release of many volatile components [33]. These components may be chemical defense factors that directly prevent insect feeding and pathogen expansion; alternatively, they may participate in plant signal transmission as alarm signals, and they can serve as antibacterial agents for pathogens [40]. Many volatile substances are distributed in the glands of plant tissues. When pathogens infect plants and damage their tissues, the glands rupture, and volatile substances are released [41]. Raffinose is a trisaccharide that is present in many plants and has antiviral and antifungal effects. Studies have shown that raffinose can activate plant defense mechanisms and increase plant resistance to pathogens [42]. Through transcriptome and metabolome analyses, we screened raffinose as an important metabolite of *G. hirsutum* in response to Verticillium wilt and identified 12 genes that were significantly correlated with raffinose; specifically, 4 genes were significantly negatively correlated with raffinose, and 8 genes were significantly positively correlated with raffinose. Additionally, guanosine can activate bursts of reactive oxygen species, callose deposition, and mitogen-activated protein kinase phosphorylation; moreover, it can activate rice resistance to sheath blight in a manner dependent on ethylene and jasmonic acid [43]. We also revealed that guanosine plays an important role in the cotton response to Verticillium wilt and identified 13 candidate genes that are significantly associated with guanosine. We also screened other volatile metabolites, such as 2-deoxyerythritol, methionine, 5-hydroxyindole-3-acetic acid, and maleamate, which play important roles in the cotton response to Verticillium wilt. However, the specific mechanisms and regulatory modules of these metabolites in regulating cotton resistance to Verticillium wilt still require further verification.

## 5. Conclusions

In this study, transcriptome and metabolome sequencing was performed on the highly susceptible Verticillium wilt variety Xinluzao 57 and the highly resistant Verticillium wilt line 192868 at different time points after inoculation with Verticillium wilt. Pathways related to amino acids and anthocyanins play important roles in cotton resistance to Verticillium wilt, and 10 volatile metabolites that play important roles in the cotton response to Verticillium wilt were identified. Candidate genes significantly associated with some metabolites were screened via WGCNA. In summary, our results provide a theoretical basis for a deeper understanding of the volatile substances involved in the response of cotton to Verticillium wilt. In the future, it will be necessary to further investigate the synthesis pathways of these important volatile metabolites, identify the genes involved in the synthesis of key enzymes, and investigate their expression and regulation to further clarify the roles of volatile substances in the defense response of cotton to Verticillium wilt.

## Data Availability

The transcriptome data used in the study have been uploaded to the NCBI database, and the transcriptome data accession number is PRJNA1234770.

## Supplementary Materials

The following supporting information can be downloaded at https://www.mdpi.com/article/10.3390/genes16080877/s1. Figure S1: Venn diagram of specific and common DEGs from Xinluzao 57 and 192868 inoculated at different time points; Figure S2: Venn diagram of specific and common DEGs within and between the materials; Figure S3: Heatmap of the metabolome correlation of upland cotton inoculated with Verticillium wilt; Table S1: All of the primers that were used in this study; and Table S2: Summary of RNA-seq analysis statistics for *G. hirsutum* samples under Verticillium wilt.
