# Supplementary figures and images for: Comparative Transcriptome and Volatile Metabolome Analysis of Gossypium hirsutum Resistance to Verticillium Wilt
Source: Genes (Basel). 2025 Jul 25;16(8):877. doi: 10.3390/genes16080877 (PMC12385826; doi:10.3390/genes16080877)

A

B

1200

8480

18400

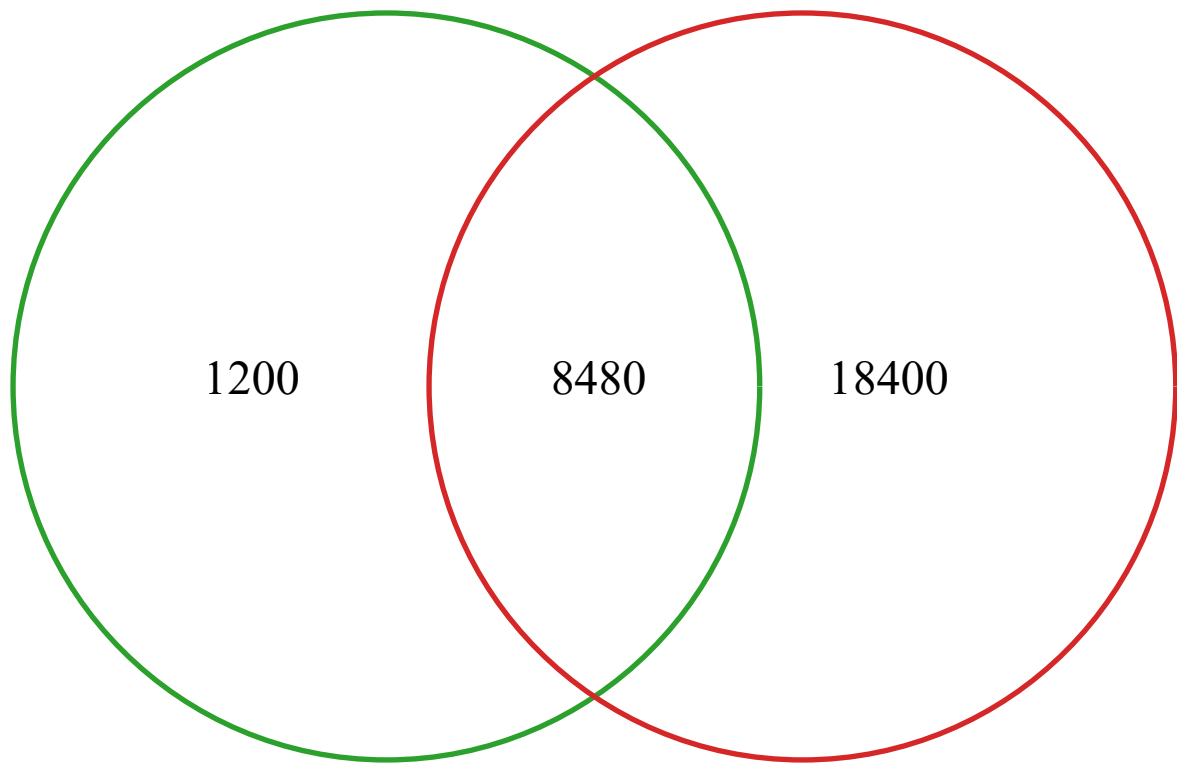

Supplement: Supplementary file 1 [file genes-16-00877-s001.zip › Supplementary File/Fig S1.pdf]

Lines inside

Between lines

6169

21911

2412

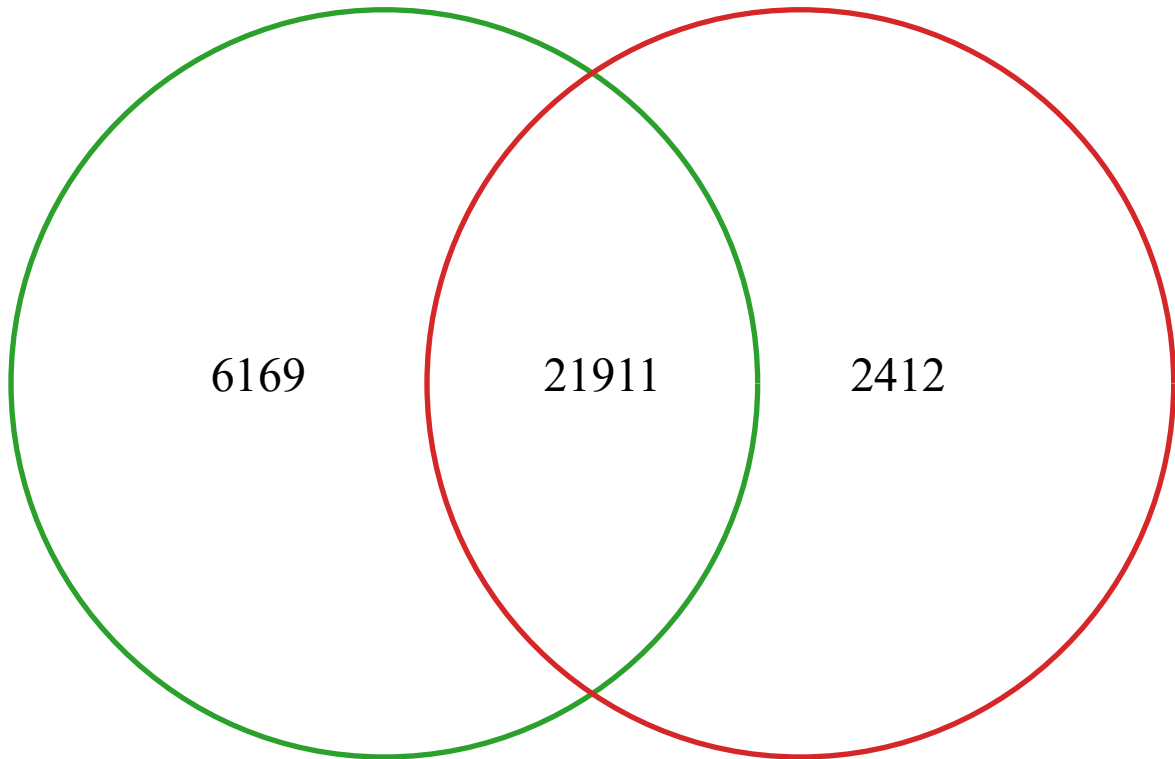

Supplement: Supplementary file 1 [file genes-16-00877-s001.zip › Supplementary File/Fig S2.pdf]

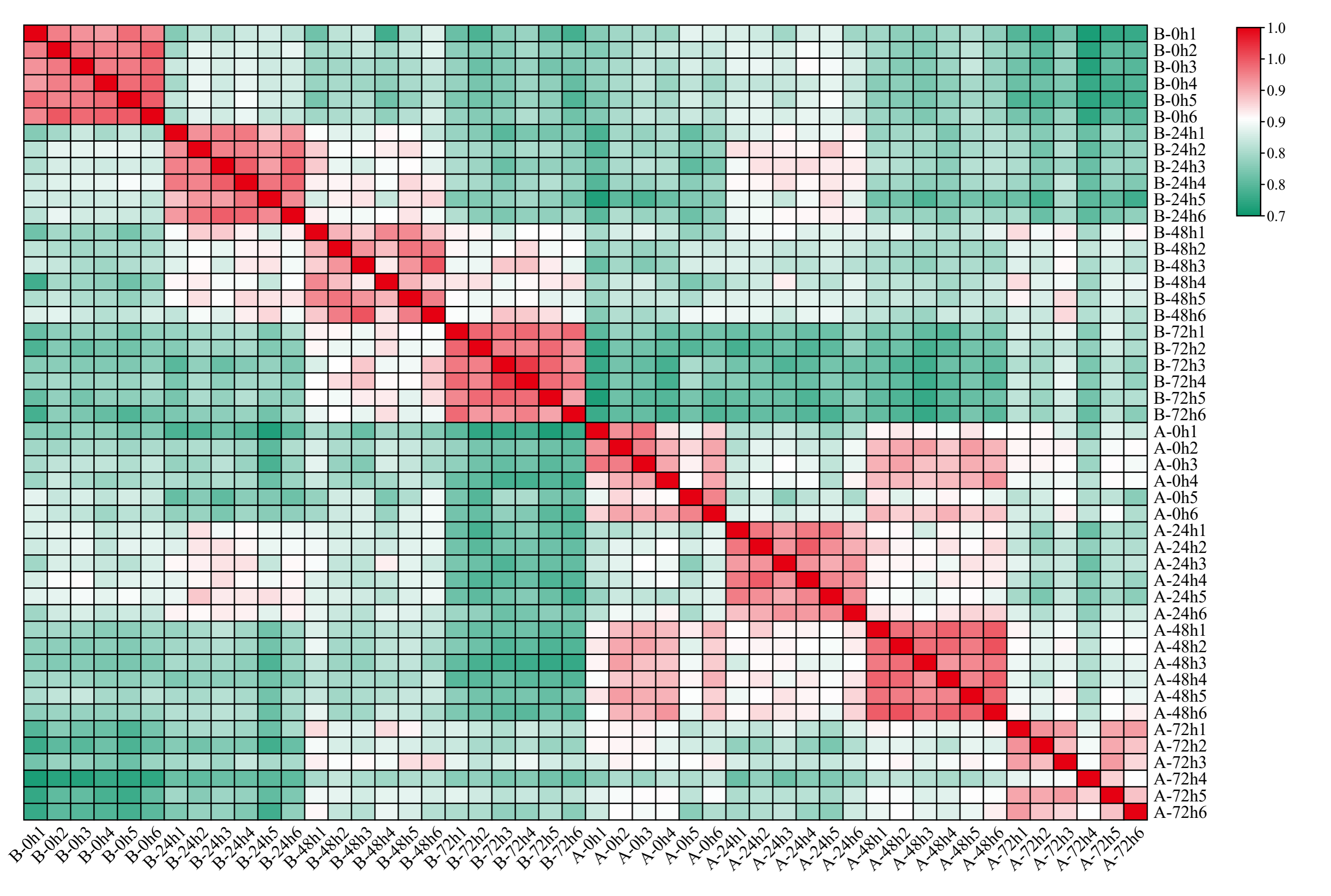

Supplement: Supplementary file 1 [file genes-16-00877-s001.zip › Supplementary File/Fig S3.pdf]
